# An Evaluation of the Fracture Resistance of Teeth with Simulated External Cervical Resorption Cavities Categorized Using Three-Dimensional Classification

**DOI:** 10.3390/jcm13082159

**Published:** 2024-04-09

**Authors:** Arzu Şahin Mantı, Özgür İlke Ulusoy

**Affiliations:** Department of Endodontics, Faculty of Dentistry, Gazi University, Bişkek (8.) Street, 1. Road, No: 8, Emek, Ankara 06490, Turkey; ilkeulusoy@gazi.edu.tr

**Keywords:** external cervical resorption, cone beam computed tomography, fracture resistance, three-dimensional classification, tricalcium silicate cement

## Abstract

(1) **Background:** External cervical resorption causes dental hard tissue destruction that may reduce the fracture resistance of affected teeth. By using a compressive strength test, this study aimed to evaluate the fracture resistance of teeth with simulated external cervical resorption cavities that have different three-dimensional classifications. (2) **Methods:** In total, 170 teeth with simulated external cervical resorptions were divided into 16 experimental groups (n = 10) and 1 control group (n = 10) based on the three-dimensional classification: 1Ap, 1Bp, 1Cp, 1Dp, 2Ap, 2Bp, 2Cp, 2Dp, 3Ap, 3Bp, 3Cp, 3Dp, 4Ap, 4Bp, 4Cp, 4Dp and a control group. Defects were restored with mineral trioxide aggregate. The fracture resistances of the samples were statistically analyzed using two-way repeated ANOVA and the Bonferroni correction for multiple comparisons at a significance level of *p* < 0.05. (3) **Results:** The lowest resistance to fracture was observed in samples with vertical height level “4” and circumferential spread of “D” (*p* < 0.001). In the groups with circumferential spreads “B”, “C” and “D”, there were significant differences between the samples with vertical height levels “1”, “2”, “3” and “4” regarding fracture resistance (*p* < 0.001). (4) **Conclusions:** The circumferential spread and vertical height of the external cervical resorption influenced the fracture resistance of the affected teeth.

## 1. Introduction

External cervical resorption is a progressive, dynamic process that affects periodontal and dental hard tissues [1]. External cervical resorption is caused by the activity of clastic cells and includes both resorptive and reparative phases [2,3]. Given its invasive and aggressive nature, external cervical resorption can lead to the destruction of root dentin in various directions [4,5]. The loss of dentine structures in the teeth with external cervical resorption reduces their fracture resistance to destructive forces such as occlusal trauma or traumatic forces, leading to the loss of the affected tooth [2,6,7].

External cervical resorption often starts in a small area in the cervical region of the tooth and spreads vertically and circumferentially through numerous channels [8,9]. Based on the size of external cervical resorption and its proximity to the root canal, Heithersay [8] formulated a classification for external cervical resorption lesions. However, this classification is only capable of describing the proximal side lesions and is not able to detect the lesions on the buccal and lingual side legions [10]. It also overlooks pulpal involvement and the circumferential spread of the external cervical resorption lesions [11]. Briefly, this classification can only two-dimensionally evaluate external cervical resorption lesions [3]. Therefore, the destructive changes that occur in the external cervical resorption process cannot be detected accurately with two-dimensional radiographical evaluation or properly categorized using the Heithersay classification [10,11].

It has been suggested that cone beam computed tomography (CBCT) may provide three-dimensional information about the actual size, location, circumferential spread and proximity of external cervical resorption lesions to the root canal [11]. According to position statements on CBCT and external cervical resorption from the European Society of Endodontology, obtaining a CBCT scan is recommended when external cervical resorption lesions are suspected during clinical examination to make a suitable diagnosis and treatment plan [12]. Patel et al. [11] proposed a new classification method for external cervical resorption lesions using CBCT. This assessment enables the three-dimensional evaluation of external cervical resorption lesions, including their height, circumferential spread and proximity to the root canal, in order to accurately determine the nature of these defects [11].

Successfully managing external cervical resorption depends on the nature and accessibility of the lesion [13]. The main treatment options for external cervical resorption are external repair of the defect with or without root canal treatment, internal repair of the defect, intentional replantation, periodical follow-ups with sensitivity tests or extraction of the tooth [13]. The external repair of external cervical resorption lesions involves curettaging the resorptive lesion to halt the resorptive process and obturation of the resorptive defect using appropriate biomaterials [13]. Dental materials such as amalgam, composite resins, glass ionomer cement and tricalcium silicate cement can be used to restore resorptive defects [14,15,16,17]. Studies indicate that periodontal reattachment is possible when biomaterials based on tricalcium silicate, such as mineral trioxide aggregate (MTA) and Biodentine, are used to restore external cervical resorption defects [18,19]. Mineral trioxide aggregate (MTA) has been suggested as a biocompatible material with good sealing ability and moisture tolerance [20,21]. It has also been reported that MTA induces periodontal healing and new cementum formation [18,22].

Root canal treatment procedures and the loss of dental hard tissues may lead to fractures in the teeth with external cervical resorption involving the pulp. Research articles in the dental literature regarding the fracture resistance of teeth with three-dimensional classifications of external cervical resorption lesions are limited [23,24]. Thus, this study aimed to evaluate the fracture resistance of teeth with simulated external cervical resorption cavities with different three-dimensional classifications using a compressive strength test. The null hypothesis was that the circumferential spread and vertical height of external cervical resorption lesions do not affect the fracture strength of the teeth.

## 2. Materials and Methods

### 2.1. Sample Selection

The in vitro experimental procedures used in this study were approved by the Gazi University Rectorate, Faculty of Dentistry, Clinical Research Ethics Committee Decision Nos. GÜDHKAEK 20.10.2022 and 2022.20/3. The teeth extracted for orthodontic and periodontal reasons were selected from the dental pool of the Faculty of Dentistry, Gazi University, Turkey. The sample size was 10 samples per group, with a power of 85% and a significance level of 5%, calculated using G* Power Version 3.1.9.6 (Franz Faul, University of Kiel, Kiel, Germany). To ensure standardization, freshly extracted human maxillary incisor teeth with a length of 20 ± 1 mm, a mesiodistal diameter of 6 ± 1 mm and a buccolingual diameter of 6 ± 1 mm were selected. The diameters of the sample teeth were measured using a digital caliper (Digital Vernier Caliper, Mitutoyo, Kawasaki, Japan). Teeth with multiple canals, calcified canals, incomplete root development, decay, cracks, fractures, resorption or restorations were excluded. Thus, 170 teeth meeting the criteria were stored in saline at room temperature before the experiment.

### 2.2. Preparation of Simulated External Cervical Resorption Cavities Based on a Three-Dimensional Classification

**Figure 1:**
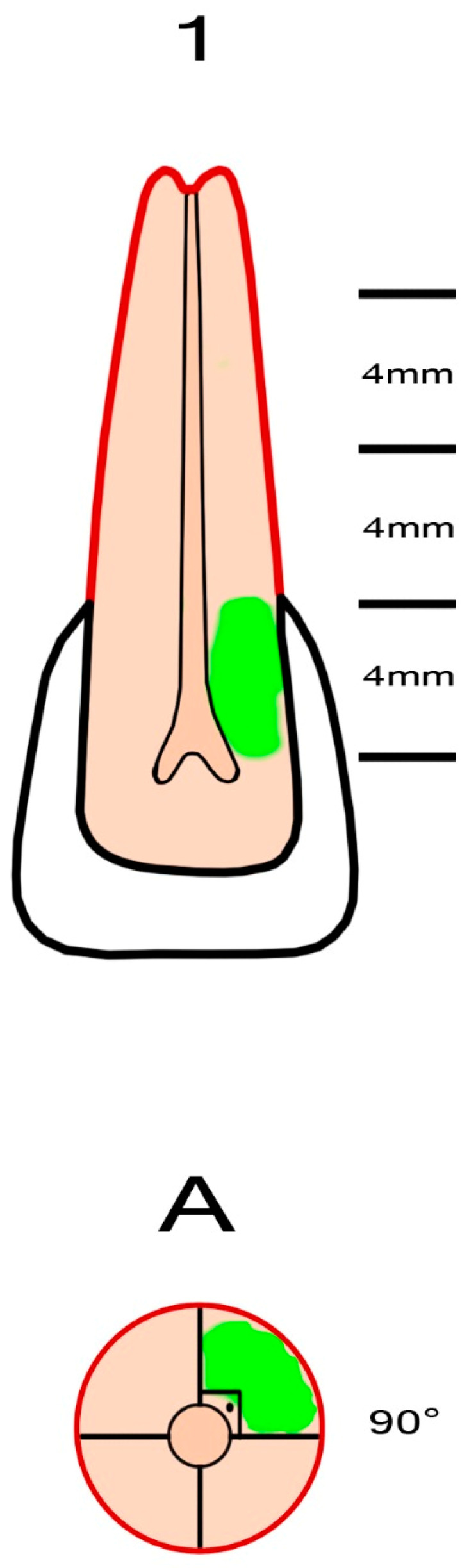
A representative figure showing the preparation of a sample with a simulated 1Ap external cervical resorption. The simulated defect with pulpal involvement has a circumferential spread of 90° and a vertical height of 3 ± 0.5 mm.

**Figure 2:**
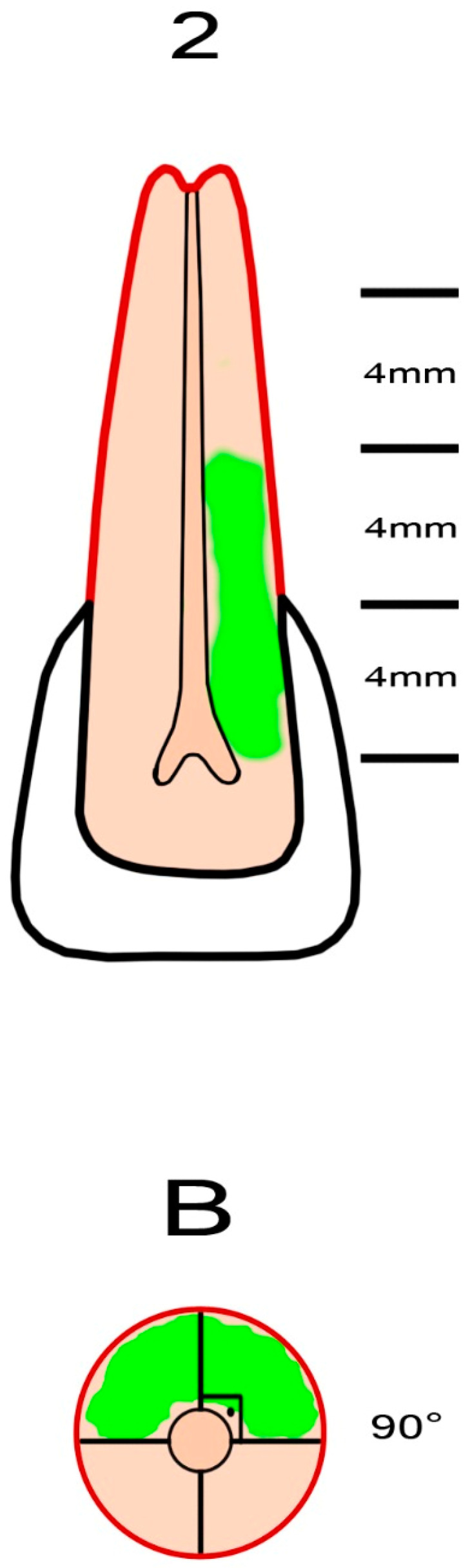
A representative figure showing the preparation of a sample with a simulated 2Bp external cervical resorption. The simulated defect with pulpal involvement has a circumferential spread of 180° and a vertical height of 7 ± 0.5 mm.

**Figure 3:**
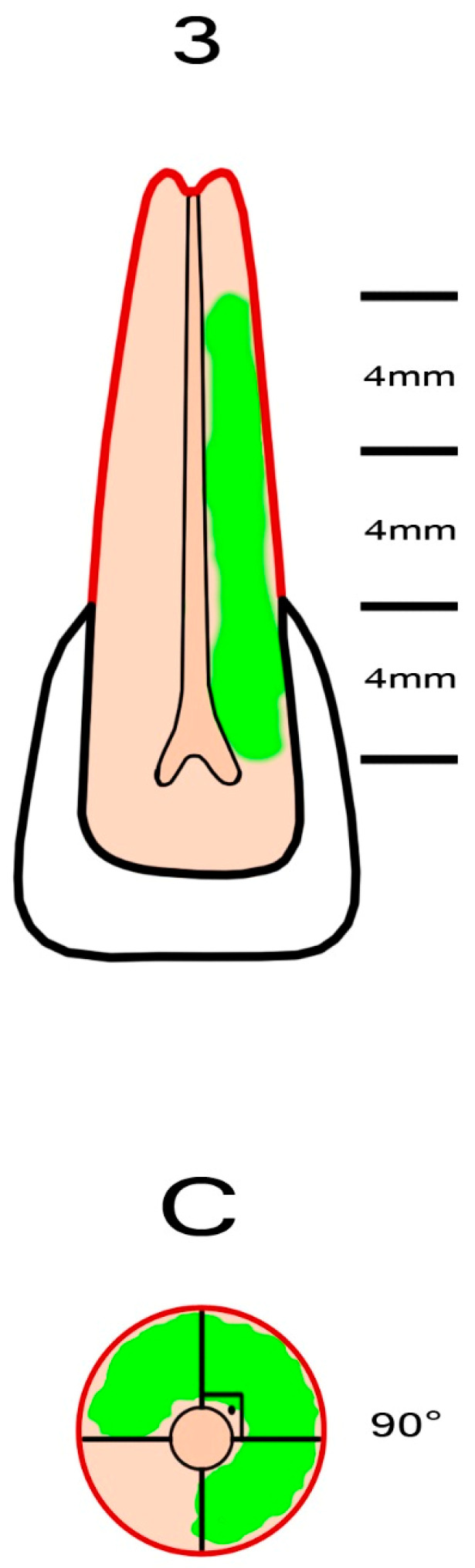
A representative figure showing the preparation of a sample with a simulated 3Cp external cervical resorption. The simulated defect with pulpal involvement has a circumferential spread of 270° and a vertical height of 11 ± 0.5 mm.

**Figure 4:**
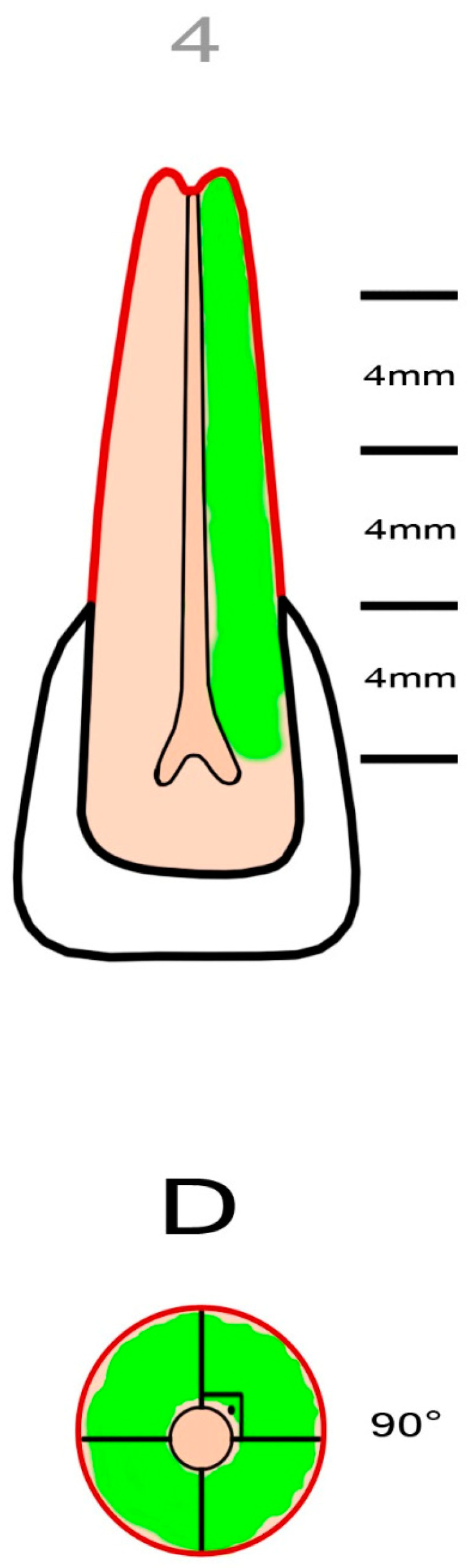
A representative figure showing the preparation of a sample with a simulated 4Dp external cervical resorption. The simulated defect with pulpal involvement has a circumferential spread of 300°–360° and a vertical height of 15 ± 0.5 mm.

External cervical resorption cavities with pulpal involvement were simulated according to the classification proposed by Patel et al. [11]. The crowns of the teeth were shortened using a diamond fissure bur, leaving a standardized tooth length of 16 ± 0.5 mm. Four equal segments representing the supracrestal part, the coronal third of the root (subcrestal), the middle third of the root and the apical third of the root were marked on the tooth using a permanent marker pen. The axial plane of the root was divided into four equal regions using a marker pen, leaving four quadrants with a radius of 3 ± 0.5 mm. To simulate the entry portal of the external cervical resorption lesion, a circular cavity with a diameter of 0.8 mm was created on the cemento-enamel junction of the sample using a diamond round bur (Dimei Royal, Dimei Dental, Anyang, Henan, China). Simulated external cervical resorption lesions with a standardized sagittal depth of 2.1 mm (mimicking external cervical resorption lesions with pulpal involvement) and varying vertical heights and different circumferential spreads were prepared. The simulated external cervical resorption cavities assessed as “A” had a circumferential spread of 90° (Figure 1). The simulated external cervical resorption cavities assessed as “B” had a circumferential spread of 180° (Figure 2). The simulated external cervical resorption cavities assessed as “C” had a circumferential spread of 270° (Figure 3). The simulated external cervical resorption cavities assessed as “D” had a circumferential spread of 300°–360° (Figure 4). The simulated external cervical resorption cavities graded as “1” (supracrestal) had a 3 ± 0.5 mm vertical height. The simulated external cervical resorption cavities graded as “2” (subcrestal) had a 7 ± 0.5 mm vertical height. The simulated external cervical resorption cavities graded as “3” (mid-third) had an 11 ± 0.5 mm vertical height. The simulated external cervical resorption cavities graded as “4” (apical third) had a 15 ± 0.5 mm vertical height.

### 2.3. Cone Beam Computed Tomography (CBCT) Evaluation

**Figure 5:**
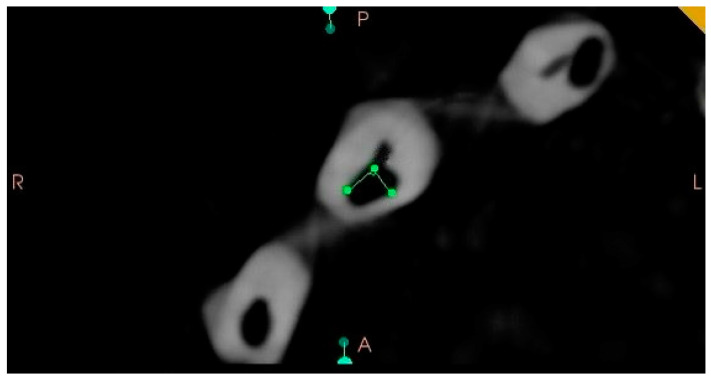
A representative CBCT image of a sample with a simulated 2Ap external cervical resorption defect. The axial slice shows the circumferential spread (90°) of the defect.

**Figure 6:**
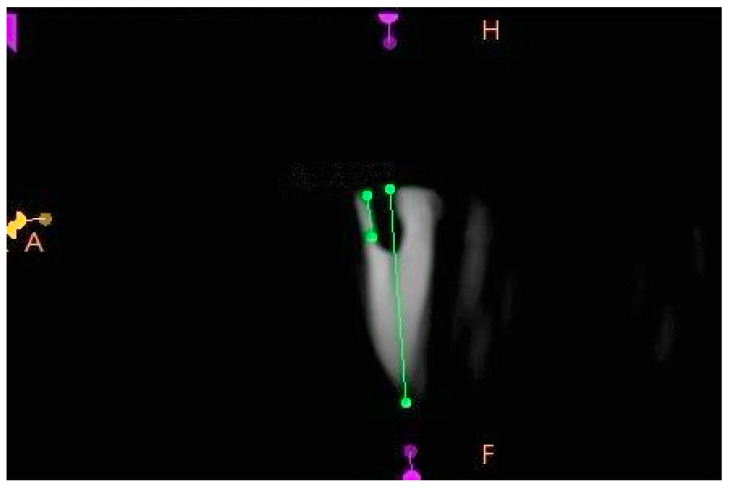
Sagittal CBCT section of a sample with a simulated external cervical resorption defect showing pulpal involvement.

**Figure 7:**
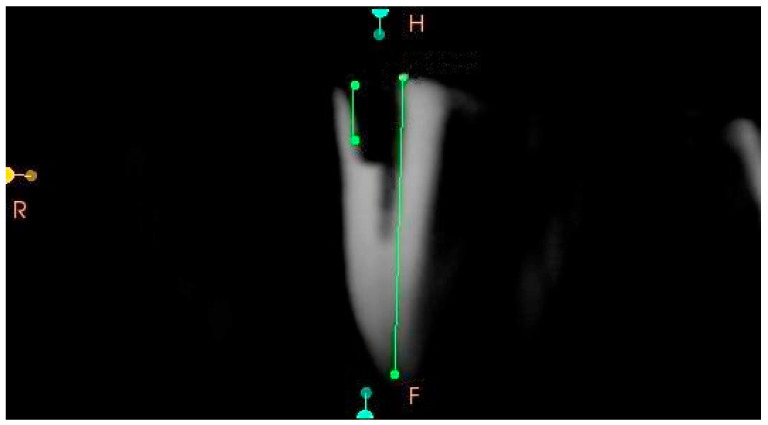
A representative CBCT image of a sample with a simulated 1Bp external cervical resorption defect. The frontal slice shows the vertical height of the external cervical resorption defect.

Following the preparation of the simulated cavities, all experimental specimens were scanned with a cone beam computed tomography (CBCT) machine (HDXwill, Dentri-s, Seoul, Republic of Korea) at a dose of 4–10 mA, an exposure time of 8–24 s and a voxel size of 200 µm to confirm the suitability of the external cervical resorption cavities for three-dimensional classification and to ensure their standardization. The CBCT scans were transferred to CS 3D Imaging software version 3.8 (Carestream Health, Rochester, NY, USA) to analyze axial, sagittal and frontal section images. The circumferential spread of the external cervical resorption lesions was measured in the axial sections of all samples and recorded (Figure 5). Proximity to the root canal in all the samples was evaluated in the sagittal, axial and frontal sections of all the specimens to confirm the pulpal involvement (Figure 6). The vertical heights of the defects were also measured from the frontal and sagittal sections of all samples and recorded (Figure 7). After evaluation of the CBCT scans, samples that did not meet the three-dimensional classification criteria were excluded and replaced with new specimens.

Ten samples with standardized tooth lengths without any cavity preparation served as controls. Thus, the 170 sample teeth were divided into 16 experimental groups (n = 10) and 1 control group (n = 10) as follows. Group 1: 1Ap; Group 2: 1Bp; Group 3: 1Cp; Group 4: 1Dp; Group 5: 2Ap; Group 6: 2Bp; Group 7: 2Cp; Group 8: 2Dp; Group 9: 3Ap; Group 10: 3Bp; Group 11: 3Cp; Group 12: 3Dp; Group 13: 4Ap; Group 14: 4Bp; Group 15: 4Cp; Group 16: 4Dp; and Group 17: intact teeth (control group).

### 2.4. Chemomechanical Instrumentation of the Root Canals

After the cone beam computed tomography procedures, root canal access cavities were created in all samples using the same round bur (Dimei Royal, Dimei Dental, Anyang, Henan, China). The working length of the samples was determined using a 15 K-file 1 mm short of the apical foramen. The root canals of 160 teeth were mechanically instrumented to the same apical size with a Dia-PT nickel–titanium file system (DPT, Dia-Dent, Cheongwon, Cheongju, Republic of Korea). During instrumentation, the samples were irrigated with 5 mL of a 2.5% sodium hypochlorite solution (Microvem, Istanbul, Turkey) using a standardized irrigation procedure. The irrigation solutions were delivered using a disposable syringe (Ultradent, South Jordan, UT, USA) and a 30-G irrigation needle (NaviTip; Ultradent, South Jordan, UT, USA) without any activation at a flow rate of 3 mL/min.

### 2.5. Filling of the External Cervical Resorption Defects

A gutta-percha cone was inserted in the root canal to avoid the extruding of the repair material. The external cervical resorption defect was restored with a tricalcium silicate-based cement (MTA Angelus, Angelus, Londrina, PR, Brazil) in all samples using an external repair technique. The root canals of all samples were then filled using a cold lateral condensation technique with gutta-percha cones (Dia Dent, Seoul, Republic of Korea) and AH Plus root canal sealer (Dentsply De Trey, Konstanz, Germany). The access cavities were sealed with conventional glass-ionomer cement (Ionoseal, Voco, Cuxhaven, Germany). The quality and standardization of the canal filling and defect repair were confirmed by periapical radiographs. The specimens were stored at a temperature of 37 °C and 100% humidity for one week to ensure setting of the filling materials.

### 2.6. Fracture Resistance Test

**Figure 8:**
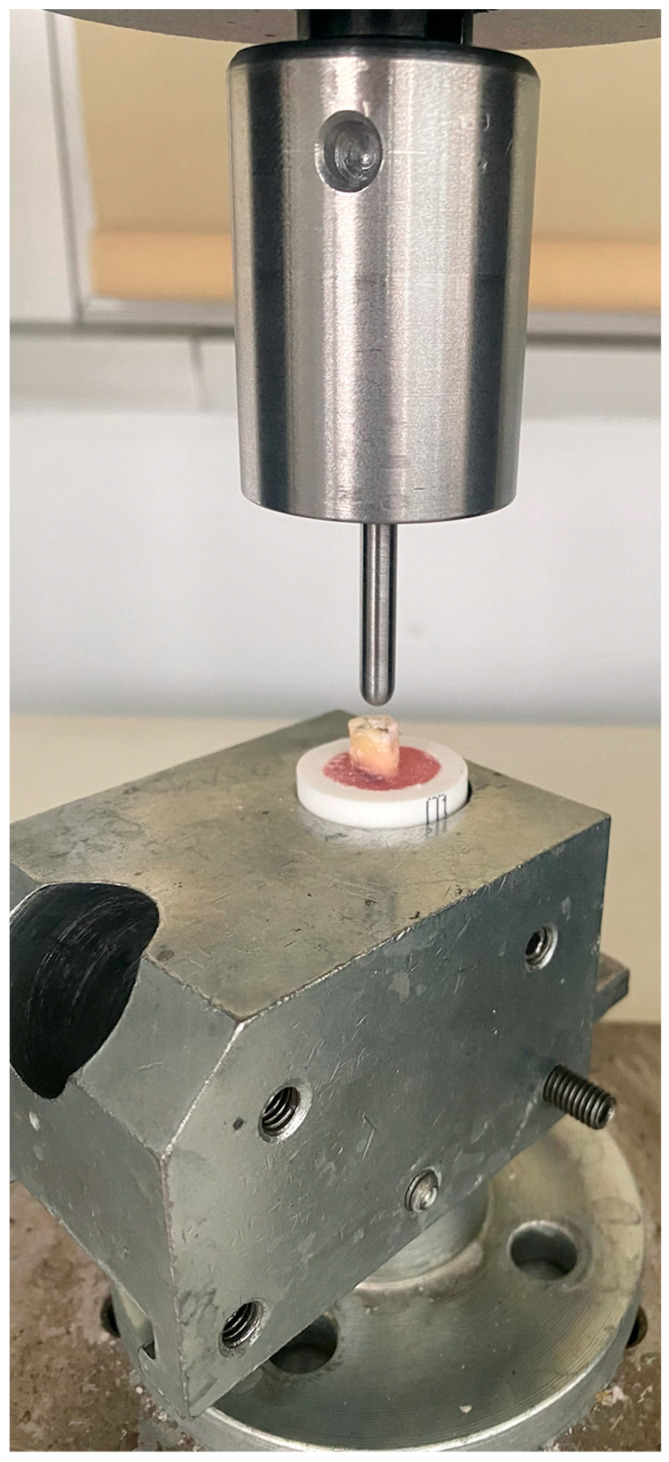
Application of compressive load on a sample.

The cement surfaces of all samples were covered with a silicone impression material of 0.3 mm (Hydrorise Light Body, Zhermack, Badia Polesine, Italy) to simulate the periodontal ligament space. The samples were then embedded vertically in self-curing acrylic resin blocks (Meliodent; Bayer UK Ltd., Newbury, UK), exposing 7 mm of the coronal parts. The blocks were positioned in the lower plate of a universal testing machine (Shimadzu AG-1; Shimadzu Corp, Tokyo, Japan) with a maximum load cell capacity of 2500 Newtons. A vertical compressive loading with a speed of 1 mm/min was applied using spherical steel balls with a diameter of 5 mm (Figure 8). The force when the fracture occurred was recorded in Newtons. Each specimen’s fracture type was evaluated after the test using a method described by Alharbi et al. [25]. Fractures that occurred within the crown were classified as crown fractures, those occurring at or above the acrylic resin level were classified as supracrestal fractures, those extending beneath the acrylic resin were classified as subcrestal fractures, and those extending along the longitudinal axis of the tooth were classified as vertical fractures [26].

### 2.7. Statistical Analysis

The data were analyzed using the Statistical Package for Social Sciences (SPSS) version 26 (IBM Corp., Armonk, NY, USA). Descriptive statistics were used for continuous variables, including the mean and standard. A two-way repeated ANOVA test was employed to compare the fracture resistances between the groups regarding the factors “vertical height and circumferential spread”. Bonferroni-corrected post hoc multiple comparison tests were used to identify significant differences. A *p* value of <0.05 was considered statistically significant.

## 3. Results

Table 1 summarizes the mean and standard deviation values for the fracture resistance of the samples with simulated external cervical resorption lesions after the mechanical test. Bonferroni corrected post hoc multiple comparison tests revealed that there were significant differences between the groups (*p* < 0.001). The control group, comprising intact samples, displayed the highest fracture resistance compared with the experimental groups (*p* < 0.001).

The lowest fracture resistance to fracture was observed in the samples with vertical height level “4” and circumferential spread “D” (*p* < 0.001). The highest fracture resistance values among the experimental groups were derived from samples with circumferential spread level “A” and vertical height level “1” (*p* < 0.001). In the groups with circumferential spreads “B”, “C” and “D”, there were significant differences in fracture resistance values between the samples with vertical height levels “1”, “2”, “3” and “4” (*p* < 0.001). In the groups classified as “1”, there were no significant differences between the samples with circumferential spread “B” and “C” (*p* > 0.05). The fracture resistance values of the samples with circumferential spreads “B” and “C” were similar in the group categorized as “3”.

The fracture types after mechanical testing are summarized in Table 2. There were no crown fractures after mechanical loading. The majority of the fractures were vertical fractures. The least commonly observed fracture type was subcrestal.

## 4. Discussion

Studies concerning the impact of dentin loss on the fracture resistance of teeth with external cervical resorption lesions are limited in the literature [23,24]. In the present study, the categorization of the simulated external cervical resorption cavities was confirmed with cone beam computed tomography (CBCT), and the fragility of teeth with differently categorized external cervical resorption defects was evaluated. To the best of our knowledge, this is the first study to evaluate the fracture resistance of teeth with different external cervical resorption cavity levels with pulpal involvement using a mechanical test and categorized using Patel’s three-dimensional classification.

Our findings demonstrated a decrease in the fracture resistance of teeth with external cervical resorption defects with an increased level of vertical height and circumferential spread, as seen in CBCT images. Thus, the null hypothesis was rejected. This result aligns with those of a previous study, which demonstrated a reduction in the fracture resistance of endodontically treated teeth with increased vertical heights [24]. That finite element study evaluated the biomechanical behavior of teeth with external cervical resorption defects with circumferential spreads of only 90° and 180° [24]. However, in the present study, all degrees of the circumferential spread of the external cervical resorption lesions were assessed with respect to their influence on the compressive strength test. Another recent study [23] compared the effect of different biomaterials on fracture resistance of teeth with external cervical resorption and used only one external cervical resorption model (2Bp as classified by Patel et al. [11]). In the present study, all of the defects were restored with the same tricalcium silicate cement, mineral trioxide aggregate (MTA), to observe the individual effect of the circumferential spread and vertical height of the external cervical resorption lesions.

Except for the samples with 90° circumferential spread, the fracture resistance values of all samples reduced as the lesion’s vertical height increased. On the other hand, external cervical resorption defects with the same vertical heights resulted in similar fracture resistance values in the samples with a circumferential spread of 180° and 270°. Considering this finding, it may be speculated that vertical height has a more significant effect than circumferential spread on the fragility of teeth with external cervical resorption to fracture.

Various restorative materials, including amalgam, composite resins, glass ionomer cement, Biodentine and MTA, have been suggested for the restoration of external cervical resorption defects [5,14,15,16,17]. Studies have indicated that tricalcium silicate cement enhances the fracture resistance of teeth by successful bonding to root dentin [26]. They also have a similar elastic modulus to that of the dentine, thus increasing the fracture resistance of the teeth [27,28]. For the above-mentioned reasons and because of its wide availability, MTA was used for the restoration of simulated external cervical resorption cavities in the present study.

Bolli et al. [29] evaluated the fracture resistances of the teeth with simulated invasive cervical resorption cavities classified using the two-dimensional Heithersay classification. They assessed the effect of different defect repair materials on the fracture resistance of teeth with Class 3 external cervical resorption cavities and found no difference between the restorative materials regarding fracture resistance. One of their findings was that the fracture resistances of intact teeth were higher compared with teeth with simulated cervical resorption cavities, which supports the findings of the present study. Although the influence of the defect repair materials on tooth fracture resistance was not evaluated in the present study, the fracture resistance values of all the sample teeth with all classification types were higher than those of the intact teeth. Considering this result and the findings of Bolli et al. [29], the loss of dental hard tissues may predominantly affect the fracture resistance of sample teeth.

In the present study, statistically significant differences were present in the fracture resistances of the teeth with external cervical resorption defects having different vertical heights after restoration using MTA. Conversely, when restored with the same biomaterial, Rajawat and Kaushik [30] reported similar stresses generated in the samples with simulated external cervical resorption defects with different vertical levels. They used finite element analysis to evaluate the stresses in the dental hard tissues affected by external cervical resorption [30]. This discrepancy may be attributable to the pulp status of the used samples, which were endodontically treated in the present study, whereas vital in the study of Rajawat and Kaushik [30]. In addition, we used a mechanical compression test method, unlike their finite element analysis. The variation in the test methods can also lead to different results within the research studies assessing fracture resistance.

Many factors could influence the results of in vitro fracture strength tests. To minimize the impact of these variables on our results, great efforts were made to standardize the parameters throughout the experiment. Standardization of the external cervical resorption cavities was achieved through the use of CBCT scans. All of the root canals were prepared using the same instrumentation technique to the same apical size. The volume and application time of the irrigation solutions and the selected irrigation technique were the same in all experimental groups, as well as the obturation materials and the filling technique.

In the present study, the majority of fractures after mechanical loading were vertical. This result is consistent with research showing that teeth with external cervical resorption having increased levels of circumferential spread and vertical height levels have a greater tendency to vertical rather than other types of fracture [23].

In actual clinical conditions, teeth can be subjected to parafunctional forces as well as functional forces. In the current research, only vertical forces with a single direction mimicking the functional forces were applied to sample teeth with external cervical resorption cavities. This issue may influence the results and can be evaluated as a limitation of this study. However, all specimens underwent fracture testing at the same angle and speed, with all standardized conditions that could affect the outcome of the fracture test being kept constant. In addition, the influence of the periodontal apparatus support can not be evaluated in the laboratory studies. In real clinical conditions, loss of periodontal attachment can be observed adjacent to the external cervical resorption defects in advanced external cervical resorption cases. This gingiva and marginal bone loss may increase the fragility of the affected tooth.

The results of the present study revealed that the advanced external cervical resorption lesions with increased vertical height and circumferential spread reduced the fracture resistance of teeth. This reduction can clinically render the teeth more fragile and reduce their survival rate. Therefore, careful evaluation of the CBCT images of the external cervical resorption lesions and their proper classification is recommended before deciding on logical management approaches aimed at long-term outcomes.

## 5. Conclusions

Three-dimensional assessment of external cervical resorption and cone beam computed tomography (CBCT) play a crucial role in the diagnosis and management of the affected teeth. Within the limitation of this in vitro study, the fracture resistance of teeth with external cervical resorption is affected by both the lesion’s circumferential spread and its height. The dentinal loss of the teeth with external cervical resorption lesions should be accurately detected using a three-dimensional classification in order to develop effective management approaches. Although great efforts were made to standardize the parameters of the present study, the behavior of the extracted teeth with simulated external cervical resorption lesions under loading may differ in actual clinical situations. Therefore, further studies in real clinical conditions and with a larger sample size are required. Additionally, finite element analysis supporting the findings derived from the mechanical tests could enhance the accuracy of the results of the present study.

## Figures and Tables

**Table 1:** The means and standard deviations of the fracture resistance values (Newton) of the samples (n = 10) with simulated external cervical resorption lesions after the mechanical test.

Vertical Height	Circumferential Spread				
	“A” (90°) n = 10 Mean (SD)	“B” (180°) n = 10 Mean (SD)	“C” (270°) n = 10 Mean (SD)	“D” (330°–360°) n = 10 Mean (SD)	*p*-Value
“1” (supracrestal) n = 10	667.25 (118.83) B,a	518.77 (86.28) B,b	468.87 (71.15) B,b	283.84 (87.67) B,c	0.001
“2” (subcrestal) n = 10	521.26 (75.13) C,a	417.84 (102.84) C,a	348.81(74.84) C,b	154.57(62.08) C,c	0.001
“3” (mid-third) n = 10	414.67 (93.76) C,a	273.68 (69.36) D,b	188.66 (50.77) D,b	73.84 (24.20) D,c	0.001
“4” (apical third) n = 10	332.52 (93.34) D,a	172.42 (44.78) E,b	95.64 (18.17) E,c	34.57 (16.13) E,d	0.001
Control group n = 10	956.67 (193.47) A,a	956.67 (193.47) A,a	956.67 (193.47) A,a	956.67 (193.47) A,a	0.001
*p*-value	0.001	0.001	0.001	0.001	

SD = standard deviation. Different lowercase letters in each row represent statistical differences between the groups classified according to the circumferential spread (Bonferroni post hoc test). Different uppercase letters in each column represent statistical differences between the groups classified according to the vertical height level (Duncan post hoc test).

**Table 2:** The number of different fracture types observed in the samples after the mechanical test.

(n = 10)	1Ap	2Ap	3Ap	4Ap	1Bp	2Bp	3Bp	4Bp	1Cp	2Cp	3Cp	4Cp	1Dp	2Dp	3Dp	4Dp	Control
Crown fracture	-	-	-	-	-	-	-	-	-	-	-	-	-	-	-	-	-
Supracrestal fracture	2	1	1	-	2	3	-	2	5	3	2	2	5	2	-	-	1
Subcrestal fracture	1	3	1	-	2	3	4	2	-	1	3	2	1	-	-	2	-
Vertical fracture	7	6	8	10	6	4	6	6	5	6	5	6	4	8	10	8	9

## Data Availability

The datasets used and/or analyzed during the current in vitro study are available from the corresponding author upon reasonable request.
